# The Effect of Optokinetic Stimulation on Perceptual and Postural Symptoms in Visual Vestibular Mismatch Patients

**DOI:** 10.1371/journal.pone.0154528

**Published:** 2016-04-29

**Authors:** Angelique Van Ombergen, Astrid J. Lubeck, Vincent Van Rompaey, Leen K. Maes, John F. Stins, Paul H. Van de Heyning, Floris L. Wuyts, Jelte E. Bos

**Affiliations:** 1 Antwerp University Research centre for Equilibrium and Aerospace (AUREA), University of Antwerp, Antwerp, Belgium; 2 Research Institute MOVE, Faculty of Behavioural and Movement Sciences, VU University Amsterdam, Netherlands; 3 Department of Otorhinolaryngology, Antwerp University Hospital, Antwerp, Belgium; 4 Faculty of Medicine and Health Sciences, University of Antwerp, Antwerp, Belgium; 5 Faculty of Medicine and Health Sciences, Department of Speech, Language and Hearing Sciences, Ghent University, Ghent, Belgium; 6 TNO Perceptual and Cognitive Systems, Soesterberg, The Netherlands; Ludwig-Maximilian University, GERMANY

## Abstract

**Background:**

Vestibular patients occasionally report aggravation or triggering of their symptoms by visual stimuli, which is called visual vestibular mismatch (VVM). These patients therefore experience discomfort, disorientation, dizziness and postural unsteadiness.

**Objective:**

Firstly, we aimed to get a better insight in the underlying mechanism of VVM by examining perceptual and postural symptoms. Secondly, we wanted to investigate whether roll-motion is a necessary trait to evoke these symptoms or whether a complex but stationary visual pattern equally provokes them.

**Methods:**

Nine VVM patients and healthy matched control group were examined by exposing both groups to a stationary stimulus as well as an optokinetic stimulus rotating around the naso-occipital axis for a prolonged period of time. Subjective visual vertical (SVV) measurements, posturography and relevant questionnaires were assessed.

**Results:**

No significant differences between both groups were found for SVV measurements. Patients always swayed more and reported more symptoms than healthy controls. Prolonged exposure to roll-motion caused in patients and controls an increase in postural sway and symptoms. However, only VVM patients reported significantly more symptoms after prolonged exposure to the optokinetic stimulus compared to scores after exposure to a stationary stimulus.

**Conclusions:**

VVM patients differ from healthy controls in postural and subjective symptoms and motion is a crucial factor in provoking these symptoms. A possible explanation could be a central visual-vestibular integration deficit, which has implications for diagnostics and clinical rehabilitation purposes. Future research should focus on the underlying central mechanism of VVM and the effectiveness of optokinetic stimulation in resolving it.

## 1. Introduction

Vestibular patients may occasionally report aggravation or triggering of their symptoms by visual stimuli. Such a presentation is typical of an entity called visual vertigo [1,2], also known as visual vestibular mismatch (VVM) [3], visually-induced dizziness [4] or space and motion discomfort[5]. Patients with VVM experience general discomfort, spatial disorientation, postural unsteadiness and dizziness due to disorienting visual environments, usually including optic flow stimulation [6]. Difficult situations for example are walking through supermarket aisles, being at crowded crossroads, watching motion pictures, scrolling across computer screens, driving a car on the highway or in tunnels. Obviously, this condition can be very debilitating and has a negative impact on quality of life and social activities since some patients develop avoidance behavior towards these situations, anxiety, phobias or even depression [7–9].

It is important to comment that VVM is highly unlikely of a sole peripheral origin, i.e. caused by an underlying vestibular deficit. Previous studies have already argued that a preceding vestibular deficit cannot solely account for the typical VVM symptoms [2] and some patients show little to no abnormalities on conventional tests of peripheral vestibular function [2,8]. In addition, a recent paper showed VVM patients to present more commonly with multiple white matter lesions than age- and gender matched controls, as seen on magnetic resonance brain imaging [10]. Although preliminary, this could suggest a contribution of white matter lesions in a part of the patients with VVM symptoms.

A possible central mechanism that could attribute to VVM is a neural representation of verticality, a mechanism that is suggested to be used by our central nervous system to differentiate between what is ‘up’ and ‘down’ with respect to the Earth-vertical in order to maintain adequate perception and postural control [11–13]. An optimal neural representation of verticality relies on a continuous interplay and integration of visual, vestibular and somatosensory information. One can expect that disorienting visual stimuli with respect to the Earth-vertical affect the neural representation of verticality by corrupting the reliability of the visual input [14]. In these situations, a healthy individual can rely predominantly on the more consistent vestibulo-proprioceptive cues in order to orient their behaviour with respect to the Earth-vertical, a process called sensory reweighting [15]. Patients with a peripheral vestibular deficit will have more difficulty to maintain adequate perception and postural control in such situations, because of the impaired vestibular information.

For VVM patients, the situation is even more complicated since these patients have an overreliance on visual cues, called visual dependency [2,8,16–18]. Visual dependency is characterized by an overreliance on visual cues instead of vestibular or proprioceptive cues for postural control, even when the visual cures might be erroneous [19]. It is known that visual dependency is distributed normally among the general population; normal individuals can be classified as highly visual dependent or not visually dependent at all [16]. However, in the case of patients, a high visual dependency may cause suboptimal perceptions. Consequently, we propose that during exposure to provocative visual stimuli, VVM patients are not capable of integrating visual, vestibular and somatosensory cues into an accurate neural representation of verticality. This difficulty could become eminent through perceptual and postural responses that rely on this representation, such as the subjective visual vertical [20], postural control [21] and visually induced motion sickness [14]. Moreover, visual roll-motion is thought to cause a tilt of the neural representation in the rotation direction [12,14,22–24], which can be derived from deviations of the subjective visual vertical and postural sway [6,8,25]. Whether this overreliance on visual cues is a primary (i.e. high visually dependent individual already prior to the later-developed peripheral vestibular pathology) or a secondary phenomenon (i.e. low or normal visually dependent individual who developed a high visual dependency as a consequence of a peripheral vestibular pathology) is not known [26].

An intervention shown to improve perceptual and postural symptoms in VVM patients is visual motion desensitization training using optokinetic stimulation [8,9,27]. Progressive, structured exposure to symptom-provoking visual motion is applied to decrease the reliance on visual cues and increase the weight of vestibulo-proprioceptive cues. Research has gained a better insight into the effects of optokinetic stimulation in VVM patients, but less is known regarding the underlying neural mechanism. If such training indeed modifies the weight given to the sensory cues, it also could improve the neural representation of verticality.

Thus, despite the growing interest and research in the use of optokinetic stimulation, little is known about the neural mechanisms underlying VVM. Moreover, no research has to our knowledge directly addressed whether motion in optokinetic stimulation is the factor causing the perceptual and postural symptoms in VVM patients. Therefore, we aimed at getting a better insight into the underlying neural mechanism of VVM by exploring perceptual and postural symptoms of VVM patients and investigated whether motion around the line of sight is a crucial condition to aggravate their symptoms. First, we hypothesized that exposure for a prolonged period of time to an optokinetic stimulus rotating around the naso-occipital axis will disrupt the neural representation of verticality more in VVM patients and therefore induces a sharper increase in their symptoms compared to healthy controls. Second, we hypothesized that visual motion is a crucial factor in aggravating symptoms in VVM patients. If a stationary “visually-loaded” stimulus is also able to evoke changes in perceptual and postural symptoms in VVM patients similar to the ones induced by visual motion, it indicates that other factors besides visual motion play a significant role. In order scrutinize these two aims, we used optokinetic stimulation as used by e.g. Guerraz et al. and Pavlou et al. [8,28] This allowed us to replicate these findings, and to obtain more insight into the neural mechanism of VVM.

## 2. Materials and Methods

### 2.1 Participants

Patients were recruited from the Department of Otorhinolaryngology at the Antwerp University Hospital. All patients underwent routine ear, nose, throat, and neuro-otological examinations, followed by specific audio-vestibular investigations and imaging when required. A detailed and systematic history was taken for each patient using the SO STONED questionnaire [29,30]. Patients were included when showing a clear pattern of VVM symptoms and triggers, based upon the visual vestibular mismatch questionnaire proposed by Mallinson [31]. Exclusion criteria were: 1) other medical conditions in the acute phase e.g. orthopaedic injury, 2) fluctuating symptoms caused by episodic vestibular disorders (e.g. Ménière’s disease) and 3) vestibular migraine. In total, 9 VVM patients were recruited (3 males, mean age(SD) 52.2(8.3) years). As age- and gender-matched controls, 9 healthy participants (3 males, mean age(SD) 51.6(6.8) years) were included. Based on the history and/or results from the audio-vestibular test battery, a peripheral vestibular disorder was identified as the likely explanation for symptom onset in 8 out of 9 patients. In total, 6 patients presented with a unilateral vestibular hypofunction (three left, four right) and one patient presented with a bilateral areflexia. One patient presented with a unilateral vestibular hyperfunction. For three of the patients with a unilateral vestibular hypofunction, vestibular neuritis could be identified as the specific etiologic diagnosis. For the other patients, a specific etiologic diagnosis could not be made since all of them were already in a chronic phase. None of the patients was assessed in an acute phase.

Ethical approval in accordance with the Declaration of Helsinki was provided by the local Ethics Committee of the University Hospital Antwerp (IRB number 14/19/220). Each participant provided a signed informed consent form. All investigations have been conducted according to the principles expressed in the Declaration of Helsinki.

### 2.2 Experimental set-up and assessment

#### 2.2.1 Stimulus

**Fig 1:**
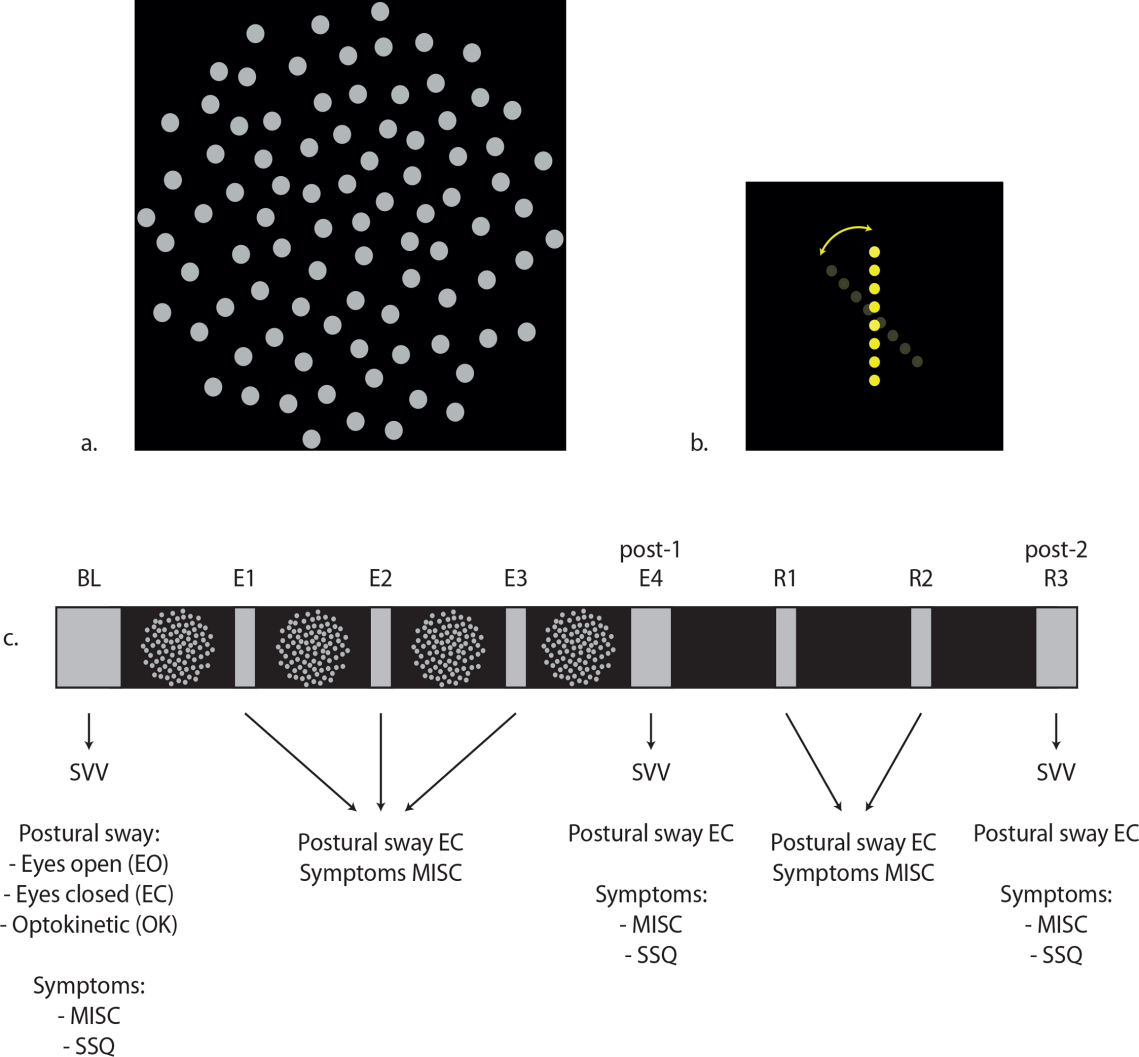
**a. The dot pattern, b. The subjective visual vertical, and c. Flow chart of the procedure.** Participants took part in two sessions following this procedure. In one session the dot pattern remained stationary during exposure (still session), while in the other session the dot pattern rotated (moving session).

Stimuli were shown on a 46-inch TV-screen (Samsung UE46ES5500) in high definition resolution (1920x1080 pixels). The screen was placed at a distance of 80cm from the participant and was adjusted in height to make sure it was eye-levelled. The stimulus was rendered using PsychToolbox 3.0 [32,33] and Matlab® R2014b. The stimulus (Fig 1A) consisted of dot pattern containing 88 non-overlapping dots, subtending a field of view of 40x40°. In case the stimulus rotated, it rotated around the naso-occipital axis with one revolution lasting 12s. The room was completely darkened, except for eyes open measurements and rest periods. In addition, exclusion of any unwanted visual Earth-fixed cues was secured by taking the following measures: 1) the edges of the TV-screen were covered with a low-reflective cardboard cover, leaving a circular viewing area with a field of view of 40x40°, 2) participants wore neutral density glasses letting through 1% of light to eliminate visual Earth-fixed cues otherwise visible by light emitted and reflected by the edges of the screen.

#### 2.2.2 Subjective visual vertical (SVV)

SVV measurements involved adjusting a rod consisting of eight small dots with a field of view of approximately 5°, during 24s to their perceived vertical (Fig 1B). The orientation of the rod was adjusted using a keypad with a roughened structure on two buttons, representing clockwise (CW) and counter clockwise (CCW) rotation. The rod was presented at an initial random offset (range: 30°-70°), either CW or CCW of the Earth-vertical. Per measurement, participants completed two trials (one with a CW offset, one with CCW). Contrary to other studies (e.g. [6,28]) we assessed the SVV in complete darkness. With this approach we excluded the direct influence of visual motion on the SVV and expected an enlarged influence of the neural processes that are affected by exposure to optokinetic stimulation, on the SVV.

#### 2.2.3 Postural sway

As a quantification of postural sway, the center of pressure (CoP) was measured on an Accugait force plate (1000Hz; Advanced Medical Technology Inc., Watertown, MA). Participants were instructed to leave their arms hanging comfortably by their sides and to position their feet 10cm apart (from the heels), with a 30° angle between the feet. During each measurement, two experimenters stood right and left at a distance of 30cm from the participant as a safety matter to prevent falls. They did not interfere or touch the participant unless necessary. Three types of measurements were performed: 1) eyes open 2) eyes closed and optokinetic. The eyes open and eyes closed measurement lasted 60s. The optokinetic measurement lasted 70s and participants had to wear the neutral density glasses. The first 10s the pattern was shown stationary, then rotated CW for 50s, and was again shown stationary for 10s. During the exposure and post-exposure phase (see below) postural sway was obtained with eyes closed. As with the SVV, with this type of measurement we aimed to exclude the direct influence of visual motion, and expected to enlarge the influence on postural sway of the neural processes affected by exposure.

#### 2.2.4 Questionnaires

Visual roll-motion is known to be provocative with respect to visually induced motion sickness (VIMS) [14,34,35]. VIMS symptoms were monitored using the simulator sickness questionnaire (SSQ) [36] and the misery scale (MISC) [37]. The SSQ scores the severity of 16 symptoms on separate 4-point scales. From these ratings, three scores were calculated representing symptom clusters of VIMS, labelled nausea, oculomotor and disorientation. A summation of the clusters results in a total score (TS). The SSQ cannot be administered in a short period of time, and therefore the MISC was included. Its employment only takes, after familiarization, a few seconds. The MISC, an 11-point scale ranging from 0 to 10 (Table 1), takes into account that symptoms of nausea are generally preceded by symptoms of oculomotor and disorienting origin [37]. The exposure was discontinued when a MISC rate of 6 or higher was reported.

**Table 1:** Misery Scale (MISC) after Bos et al. [37].

Symptom	Severity	Score
No problems		0
Uneasiness (no typical symptoms)		1
Dizziness, warmth, headache, stomach awareness, sweating, and other symptoms	Vague Slight Fairly Severe	2 3 4 5
Nausea	Slight Fairly Severe	6 7 8
Retching		9
Vomiting		10

### 2.3 Protocol

Each participant completed two sessions (still and moving) in a randomized and counterbalanced order on the same day. In the first session participants signed the informed consent form. Each session consisted of three phases: 1) a baseline phase, 2) an exposure phase and 3) a post-exposure phase (Fig 1C). The protocol was identical for both sessions.

#### 2.3.1 Baseline phase

First, participants reported a MISC rate and filled out the SSQ. Second, an SVV measurement was obtained. Thirdly, three postural sway measurements were conducted: an eyes open, eyes closed and optokinetic measurement.

#### 2.3.2 Exposure phase

The exposure phase consisted of 4x5min exposure. During each block, the stimulus either was shown stationary (still session) or rotated (moving session), the rotation direction alternated after 5 revolutions, resulting in 5 reversals of direction per block. After every block, participants had to report a MISC rate and an eyes closed postural sway measurement was obtained (Fig 1C). After completion of 4 exposure blocks (E4), defined post-1, also an SVV measurement was obtained and the SSQ was filled out.

#### 2.3.3. Post-exposure phase

The post-exposure phase consisted of 3x5min rest. In analogy with the exposure phase, a MISC rate and an eyes closed postural sway measurement were obtained (Fig 1C). After the third post-exposure block (R3), defined post-2, participants again performed an SVV measurement and filled out the SSQ next to an eyes closed postural sway measurement and the MISC rate.

### 2.4 CoP data analysis

CoP data were analyzed using Matlab® R2014b. CoP time series were filtered with a 2^nd^ order low-pass Butterworth filter with a cut-off frequency of 5Hz. To avoid onset-effects and to only study the effect of measurement types (eyes open, eyes closed, optokinetic) obtained at baseline, the first 10s of each time series were discarded and for the optokinetic time series also the last 10s were discarded. To study the effect of prolonged exposure, the first 5s were discarded from all eyes closed measurements. From the filtered CoP time series, we calculated the sway path length (SPL), defined as the length that the CoP travelled in one measurement. Second, by showing roll-motion around the naso-occipital axis, the largest displacements were elicited in the mediolateral direction, which were quantified with the standard deviation in mediolateral direction (SDML).

### 2.5 Statistical analyses

Separate univariate mixed model ANOVA’s were used to statistically test SPL, SDML and SVV data. Regarding baseline measurements, to investigate effects of the type of measurement (eyes open, eyes closed, optokinetic), group (patients and controls) and session (still and moving) a three-way mixed model ANOVA was conducted with measurement type and session as within-subject factors and group as between-subject factor. To study effects of the optokinetic stimulus in the exposure- and post-exposure phase we included for ease of analysis three essential measurement moments, being BL, post-1 and post-2. To investigate effects of session (still and moving), measurement moment (BL, post-1, post-2) and group (patients and controls) a three-way mixed model ANOVA was performed, with measurement moment and session as within-subject factors and group as between-subject factor. Significant effects were followed up with difference contrasts. All variables appeared to meet the assumption of normality as checked with Shapiro-Wilk tests. If the assumption of sphericity was violated, a Greenhouse-Geisser correction was applied.

Changes in MISC rates and SSQ total scores were examined using separate Friedman tests for each session (still and moving) and group (patients and controls). Significant results were followed up with Bonferroni corrected Wilcoxon Signed Rank tests. To study differences between sessions within each group, again Wilcoxon Signed Rank tests were used. Differences between groups were investigated using Mann-Whitney U tests.

## 3. Results

### 3.1 Subjective visual vertical

SVV deviations were not significantly different between the groups. Moreover, the SVV obtained after prolonged exposure to optokinetic stimulation was not significantly different from the SVV obtained after exposure to the stationary pattern for both groups. No significant interaction between group and session was observed.

### 3.2 Postural sway

#### 3.2.1 Baseline

**Fig 2:**
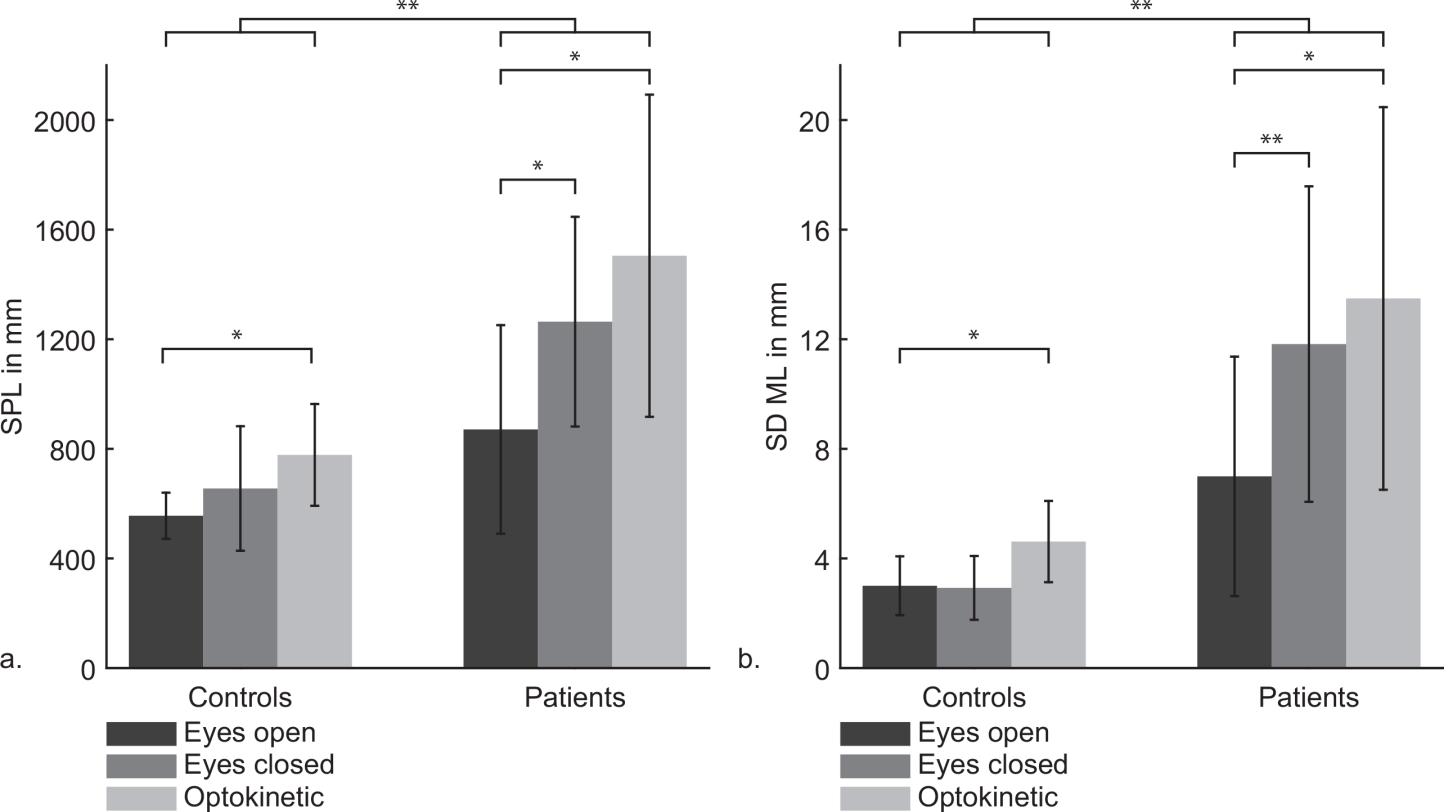
**a. The sway path length (SPL) in mm (±SEM) and b. Standard deviation in mediolateral direction (SDML) in mm (±SEM) for both groups and all measurement types.** VVM patients always exhibited a higher SPL and SDML than healthy controls. VVM patients were significantly more affected by visual deprivation (i.e. eyes closed versus eyes open) than healthy controls. Significant differences at *p <* .*05*, *p <* .*01* and *p <* .*0001* are indicated with *, **, and ***, respectively. EC: eyes closed; EO: eyes open; OK: optokinetic.

Baseline postural sway in the still and moving sessions did not differ, therefore Fig 2 shows the SPL and SDML averaged over the still and moving sessions. Patients exhibited a significantly larger SPL and SDML than controls irrespective of the measurement type, *F(*1,15) = 15.30, *p* = .001, *η*_*p*_^*2*^ = .51 and *F*(1,15) = 17.72, *p* = .001, *η*_*p*_^*2*^ = .54, respectively. The SPL and SDML also differed significantly between the three measurements types, *F(*1.42,21.24) = 16.37, *p* < .0001, *η*_*p*_^*2*^ = .52 and *F*(2,30) = 11.81, *p* < .0001, *η*_*p*_^*2*^ = .44, respectively. Difference contrasts revealed that, as anticipated, the SPL and SDML were significantly larger whilst exposure to rotation (optokinetic) compared to the eyes closed and eyes open measurements (*p* = .002 and *p* = .001, respectively). Moreover, the SPL and SDML were also more elevated in the eyes closed measurement compared to the eyes open measurement (*p* < .0001 and *p* = .011, respectively). Most interestingly was the significant interaction between group and measurement type, *F*(2,30) = 3.99, *p* = .029, *η*_*p*_^*2*^ = .21 and *F*(2,30) = 5.68, *p* = .008, *η*_*p*_^*2*^ = .28 for the SPL and SDML, respectively. Difference contrasts combined with ANOVAs separate for each group unveiled that the SPL and SDML in VVM patients were significantly more affected by closing the eyes than healthy controls (Tables 2 and 3). However the optokinetic stimulus caused a significant increase of the SPL and SDML compared to the eyes open measurement in both groups (Tables 2 and 3).

**Table 2:** Difference contrasts for the interaction between group and measurement type separated for each group for SPL.

*Source*	*Df*	*F*	*η*_*p*_^*2*^	*p*
SPL				
***Patients***				
EC vs EO**	1	21.01	.75	.003
OK vs EC+EO*	1	7.48	.52	.029
***Controls***				
EC vs EO	1	2.45	.23	.156
OK vs EC+EO*	1	11.22	.58	.010

* p < .05

** p < .01

**Table 3:** Difference contrasts for the interaction between group and measurement type separated for each group for SDML.

*Source*	*Df*	*F*	*η*_*p*_^*2*^	*p*
SDML				
***Patients***				
EC vs EO*	1	8.14	.54	.025
OK vs EC+EO*	1	7.67	.52	.028
***Controls***				
EC vs EO	1	.035	.004	.856
OK vs EC+EO**	1	11.81	.596	.009
Error Patients	7			
Error Controls	8			

* p < .05

** p < .01

#### 3.2.2 Exposure

**Fig 3:**
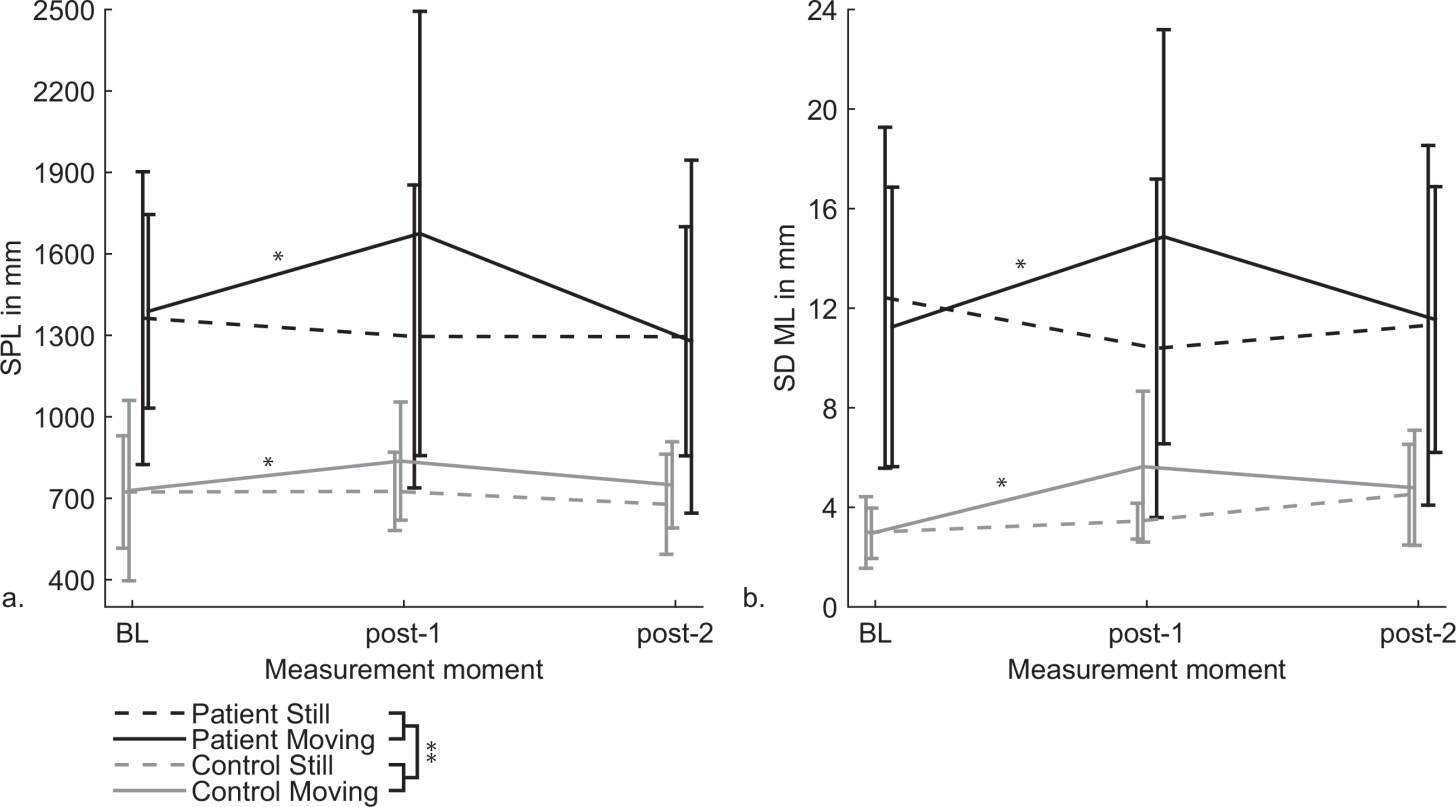
**a. The sway path length (SPL) in mm (±SEM) and b. Standard deviation in mediolateral direction (SDML) in mm (±SEM) for both groups and sessions.** VVM patients always exhibited a higher SPL and SDML than healthy controls. Both VVM patients and controls had a higher SPL and SDML due to exposure to optokinetic stimulation compared to eyes open. Significant differences at *p <* .*05* are indicated with *.

Fig 3 depicts the SPL and SDML for both groups and sessions obtained before exposure, directly after exposure and at the end of the session. Both the SPL and SDML were significantly larger for the VVM patients compared to the healthy controls, *F*(1,15) = 12.41, *p* = .003, *η*_*p*_^*2*^ = .45 and *F*(1,15) = 14.87, *p* = .002, *η*_*p*_^*2*^ = .50, respectively. Based on the hypotheses a significant interaction was expected between group and measurement, however the large main effect of group may have impeded this interaction. Only for the SPL a main effect of session was found, i.e. the SPL in the moving session was higher compared to the still session, *F*(1,15) = 5.61, *p* = .032, *η*_*p*_^*2*^ = .27. The SPL, as well as the SDML were affected by the interaction of the measurement moments with the sessions, but was only significant for the SDML, *F*(2,30) = 2.95, *p* = .068, *η*_*p*_^*2*^ = .16 and *F*(2,30) = 7.20, *p* = .003, *η*_*p*_^*2*^ = .32. Difference contrasts revealed that the SPL and SDML were significantly elevated directly after exposure (post-1) in the moving session, but not in the still session, *F*(1,15) = 8.04, *p* = .013, *η*_*p*_^*2*^ = .35 and *F*(1,15) = 13.08, *p* = .003, *η*_*p*_^*2*^ = .47.

### 3.3 Questionnaires

**Fig 4:**
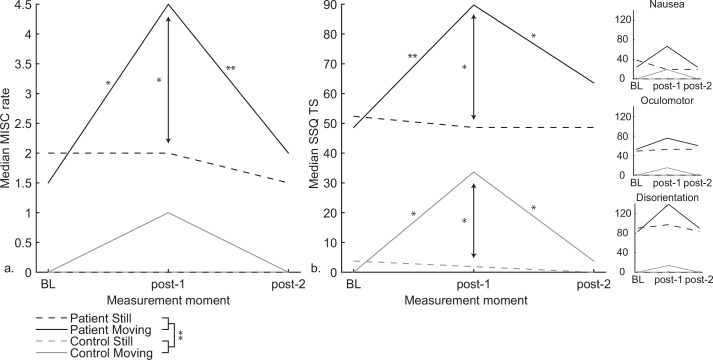
**a.** Median MISC rate and **b.** Median SSQ total score (TS) with the symptom cluster scores depicted in the inset. VVM patients always reported more severe symptoms than controls. Both VVM patients and controls reported an increase in symptom severity due to exposure to optokinetic stimulation. Significant differences at *p <* .*05 and p <* .*01* are indicated with * and **, respectively

MISC rates reported by the VVM patients were significantly higher than the MISC rates reported by healthy controls at all measurement moments, all *p*’s < .004 (Fig 4A). MISC rates did not change in the still session, but were significantly affected in the moving session for both VVM patients and controls, χ^2^(2) = 9.29, *p* = .01 and χ^2^(2) = 13.00, *p* = .002, respectively. Further analysis revealed that MISC rates reported directly after exposure to the rotating pattern (post-1) were significantly elevated compared to before (BL) exposure and after rest (post-2) only for VVM patients (Table 4). Moreover, comparison of the still session to the moving session revealed that only patients reported significantly higher MISC rates after exposure to the optokinetic stimulus compared to the MISC rates reported after exposure to the stationary pattern (Table 4).

**Table 4:** Wilcoxon signed rank tests as a follow-up for significant Friedman tests for the MISC.

*Factors*	*Z*	*p*
MISC		
***Patients***		
Moving session; BL–Post1*	2.39	.016
Moving session; Post1 –Post2**	2.56	.008
Moving session; BL–Post 2	.535	.750
Moving vs Still; BL	.531	.719
Moving vs Still; Post 1*	2.214	.031
Moving vs Still; Post 2	.447	1.00
***Controls***		
Moving session; BL–Post1	2.06	.063
Moving session; Post1 –Post2	2.12	.063
Moving session; BL–Post 2	1.41	.500
Moving vs Still; BL	1.00	1.00
Moving vs Still; Post 1	2.07	.063
Moving vs Still; Post 2	1.41	.500

* p < .05

** p < .01

SSQ TS showed similar results to the MISC rates (Fig 4B). SSQ TS were at all measurement moments significantly higher for VVM patients than for healthy controls, (Table 5). In the still session the SSQ TS did not significantly change, but was significantly increased in the moving session for both patients and controls, χ^2^(2) = 9.07, *p* = .011 and χ^2^(2) = 12.19, *p* = .002, respectively. Follow-up tests revealed that for patients as well as controls the SSQ TS were significantly elevated directly after exposure to the optokinetic stimulus (post-1) compared to baseline scores and scores reported after rest (post-2), (Table 5). Comparison of the still session to the moving session, however, revealed that patients and controls reported significantly higher SSQ TS after exposure to the optokinetic stimulus. Investigation of the symptom clusters of the SSQ (Fig 4B insets) revealed that patients reported mostly symptoms of disorienting origin, followed by oculomotor and nausea symptoms in the moving session. Controls reported minor symptoms with equal scores on all three subscales.

**Table 5:** *Wilcoxon signed rank tests as a follow-up for significant Friedman tests for the SS*Q.

*Factors*	*Z*	*p*
SSQ		
***Patients***		
Moving session; BL–Post1**	2.52	.008
Moving session; Post1 –Post2*	2.37	.016
Moving session; BL–Post 2	1.20	.250
Moving vs Still; BL	1.19	.266
Moving vs Still; Post 1*	2.03	.047
Moving vs Still; Post 2	1.33	.195
***Controls***		
Moving session; BL–Post1*	2.24	.023
Moving session; Post1 –Post2*	2.37	.016
Moving session; BL–Post 2	.412	.813
Moving vs Still; BL	.954	.438
Moving vs Still; Post 1*	2.38	.016
Moving vs Still; Post 2	1.51	.250

* p < .05

** p < .01

## 4. Discussion

The present study was designed to provide more insight into the effects of optokinetic stimulation on the SVV, postural sway and visually induced symptoms among VVM patients and healthy controls. We hypothesized visual roll-motion to be a crucial factor in the causation of SVV deviations, increased postural sway, and VVM symptoms. We also expected VVM patients to show more severe changes due to prolonged exposure to optokinetic stimulation compared to healthy controls, due to suboptimal sensory signals, secondary to a vestibular insult of peripheral origin.

### 4.1 Subjective visual vertical

For SVV measurements, which were proposed to reflect changes in the neural representation of verticality, we did not find any differences between patients and controls or due to prolonged exposure to optokinetic stimulation. In other words, the proposed after-effect of the optokinetic stimulus was not strong enough to significantly affect the SVV. One possible explanation for this lack of difference could be that the exposure duration to the optokinetic stimulus was not long enough to significantly affect the neural representation of verticality. Further research could help in identifying how long the exposure should take to affect the SVV, and thus the neural representation of verticality. In addition, if the neural representation, and thereby the SVV, would be more affected by visual roll-motion in VVM patients compared to healthy controls, this would also be visible by incorporating SVV measurements during exposure to roll-motion, as performed by e.g. Guerraz et al. [8]. The fact that many of the VVM patients had an underlying peripheral vestibular pathology in the past did not have an influence on the SVV deviations, since this test is more appropriate to detect acute unilateral peripheral vestibular dysfunction, and the current patients were all tested in a chronic state. In addition, it would be interesting to assess the subjective tactile vertical in future studies. Changes in the subjective tactile vertical could provide a more detailed insight into the assumed underlying central deficit in these patients [38].

Another possible explanation could be, assuming that the optokinetic stimulation was strong enough, that VVM is not restricted to visuo-vestibular functions but that it also involves sensorimotor control schemes. This has already been shown to be the case by several neurophysiological studies in patients with phobic postural vertigo [39,40]. Future studies should look into this in order to get a better understanding of the underlying mechanisms of postural control in VVM.

### 4.2 Postural responses

Comparing VVM patients with healthy controls, patients always swayed more than controls irrespective of the measurement type (eyes open, eyes closed, optokinetic). Secondly, VVM patients’ postural sway was significantly increased when deprived of visual cues, while this was not the case for controls. In the absence of visual cues, participants need to rely predominantly on vestibulo-proprioceptive input information in order to control posture. Since most of our VVM patients had an underlying peripheral vestibular pathology this vestibulo-proprioceptive signal is suboptimal, resulting in increased postural sway. If the vestibular cues itself are incorrect, it would indicate a peripheral vestibular cause, whereas an incorrect sensory reweighting, i.e. visual dependency, would point towards a central problem. Both phenomena will exert a larger influence on postural control due to loss of visual cues and therefore could explain the large increase in postural sway obtained with eyes closed in VVM patients. In a study by Guerraz et al. [8] no differences between eyes open and eyes closed postural sway were found for VVM patients, which is probably related to the fact that patients in their study presented with much less peripheral vestibular deficits in comparison to our VVM patients.

Another important finding is that postural sway significantly increased during exposure to optokinetic stimulation compared to eyes open in both patients and controls. This demonstrates that roll-motion around the naso-occipital axis can increase postural sway in a healthy participant, although to a lesser extent than in a VVM patient. The observation that patients revealed more variable and larger postural sway increases than controls due to exposure to such full-field motion (engaging pretectal visual pathways), may implicate they either have an abnormality in the perception of visual motion itself or an impaired central integration (engaging cerebellar pathways). In the same light, a preliminary study showed that VVM patients exhibit differences to healthy controls in gray and white matter structures such as the primary visual cortex and the cerebellar pathways [41], which should be further investigated and linked to the current results.

Finally, exposing both groups to prolonged optokinetic stimuli resulted in a significant increase of postural sway, which was not visible when a stationary stimulus was used. This proves that visual motion is a crucial factor in increasing postural sway. The results also suggest a common neural origin that modulates both postural sway and visually induced symptoms. Moreover, these findings substantiate the hypothesis that VVM symptoms are aggravated by visual motion [42].

### 4.3 Subjective responses

A comparison of both groups showed that the MISC and SSQ scores were always significantly higher for VVM patients than for controls. We also observed that MISC rates and SSQ TS were significantly higher after exposure to roll-motion compared to before exposure, while no such increase was found in the still session. Moreover, these findings are in accordance with the postural responses, linking subjective and objective symptoms in these patients. They also indicate that roll-motion is essential for the aggravation of visually evoked symptoms, which is in line with an earlier study [42]. The observation that roll-motion is necessary to aggravate VVM symptoms also suggests that roll-motion is essential for training and rehabilitation purposes, which is in accordance with earlier findings [6].

The MISC revealed that only VVM patients reported significantly higher scores after optokinetic stimulation compared to MISC rates provided after exposure to a stationary stimulus. Although controls reported higher rates after optokinetic stimulation, this increase was not significantly different from the rates reported in the still session. As expected and already suggested by Guerraz and colleagues [8], this highlights the fact that the symptoms that VVM patients experience are not comparable to symptoms experienced occasionally by healthy individuals in disorienting visual environments, i.e. healthy individuals may experience symptoms similar to the symptoms of VVM patients; however, the severity of these symptoms is much lower compared to the severity of symptoms experienced by VVM patients.

### 4.4 Limitations, considerations and future studies

The current study had several limitations. A first limitation is the lack of a “vestibular control group”. Ideally, this would be patients with a clear peripheral vestibular deficit but with absence of VVM symptoms (assuming an adequate neural compensation process) or a group of vestibular patients with a bilateral peripheral deficit (assuming no vestibular input whatsoever). Also, we did not take the contribution of psychogenic components into account. It has been shown repeatedly that there is a high comorbidity of psychiatric and anxiety disorders in vestibular patients in general [43]. However, in a previous study with VVM patients, no effect of anxiety trait was found when compared to normal controls and labyrinthine-defective subjects [8]. On the contrary, more previous studies have shown that improvements in VVM symptoms are related to improvements in psychological and anxiety-related symptoms, suggesting a link between VVM and psychiatric disorders [28]. A recent paper investigating anxiety in individuals with VVM and vestibulopathy also showed that VVM should be considered in anxious patients with imbalance deficits [44]. Furthermore, recent studies in acrophobia and visual height intolerance have also shown that anxiety can influence the perception of visuo-vestibular signals and self-recognition of body sway and thus concurrently affect the motor output itself [45–47]. It is even suggested that dual tasking could lead to postural improvements in individuals suffering from visual height intolerance. The authors suggest that these postural improvements could be explained by the fact that mental distraction (in the form of cognitive dual tasking) leads to anxiety reduction [46,47]. Therefore, future studies should be set up to specifically investigate the psychological involvement in VVM patients and in addition, to investigate the impact of anxiety on visuo-vestibular exploration and motor behavior, to get more insight into this matter.

It is also important to make a distinction between VVM [31], phobic postural vertigo (PPV) [48,49] and chronic subjective dizziness (CSD) [50], where possible. Although all three disorders might resemble each other very much, they are seen as different entities. VVM, PPV and CSD are often mistaken since all are associated (either primary or secondary) with psychiatric disorders such as anxiety, depression, panic attacks and even obsessive-compulsive personality traits [44,51,52]. PPV is characterized by a persistent sense of unsteadiness or postural imbalance and recurrent dizziness attacks [48,49,51]. The prevalence of PPV is reported to be quite high [53] and it is even considered the most common form of dizziness in a neuro-vestibular unit in middle-aged patients [54]. Furthermore, PPV patients do not suffer specifically from visually-induced dizziness [55], i.e. dizziness arising after exposure to certain provoking visual stimuli (examples of these are as mentioned above supermarkets, busy traffic and watching action movies among others), while this is an important and specific feature of VVM [1,31]. CSD is characterized by persistent non-vertiginous dizziness and unsteadiness. Important to note is that CSD can also present with a hypersensitivity to self-motion and/or visual stimuli [50]. CSD is reported to be the second most frequent common cause of dizziness in tertiary neuro-otology clinics that know of its existence. It should be kept in mind however that a comorbidity between VVM and CSD can occur [56]. Recently, a new related entity has been suggested: persistent postural-perceptual dizziness (PPPD). In definition, PPPD patients present with a variety of visual, motor and postural symptoms [57–59]. It is possible that PPPD presents in different subtypes, e.g. a postural subtype or a visual subtype, depending on the overriding symptoms in an individual patient. If future research could support valid subtypes, this could mean that PPPD might eventually become an overarching entity for VVM, PPV and CSD.

### 4.5 Clinical relevance

Our findings confirm the results from previous studies and therefore add to the robustness of these findings in the complex syndrome that is called visual vestibular mismatch. Our results propose that a measurement such as postural sway could be used as a marker to evaluate the progress of rehabilitation programs, however gait, subjective symptoms and triggers should also be kept in mind. One of the therapeutic possibilities is the use of optokinetic stimuli in order to desensitize these patients to visual stimuli and to facilitate the sensory reweighting process [6,28]. Our study corroborates this, as we showed that motion is crucial in elucidating both subjective and postural symptoms. A rehabilitation program based on disorienting motion around the naso-occipital axis can therefore provide the necessary “challenges” needed to train and desensitize these patients. It is however crucial to develop a rehabilitation scheme that is graded, repetitive and individually adapted[6]. All of the above has important implications for patients with visual vestibular mismatch syndrome, a debilitating, undervalued and rarely studied condition.

### 4.6 Conclusion

In summary, this study showed that visual vestibular mismatch patients experience postural and perceptual symptoms in disorienting moving visual environments. This has important implications for diagnostic decision-making and for the evaluation and set-up of clinical rehabilitation programs. Further studies should investigate the central mechanism proposed to underlie VVM symptoms and the effectiveness of optokinetic stimulation as a rehabilitation method to resolve it.

## Supporting Information

S1 File Dataset underlying the presented data. (ZIP)
